# Identifying the Computational Requirements of an Integrated Top-Down-Bottom-Up Model for Overt Visual Attention within an Active Vision System

**DOI:** 10.1371/journal.pone.0054585

**Published:** 2013-02-20

**Authors:** Sebastian McBride, Martin Huelse, Mark Lee

**Affiliations:** 1 Department of Physiology, Development and Neuroscience, University of Cambridge, Cambridge, United Kingdom; 2 Department of Computer Science, Abersytwyth University, Aberystwyth, United Kingdom; The University of Plymouth, United Kingdom

## Abstract

Computational visual attention systems have been constructed in order for robots and other devices to detect and locate regions of interest in their visual world. Such systems often attempt to take account of what is known of the human visual system and employ concepts, such as ‘active vision’, to gain various perceived advantages. However, despite the potential for gaining insights from such experiments, the computational requirements for visual attention processing are often not clearly presented from a biological perspective. This was the primary objective of this study, attained through two specific phases of investigation: 1) conceptual modeling of a top-down-bottom-up framework through critical analysis of the psychophysical and neurophysiological literature, 2) implementation and validation of the model into robotic hardware (as a representative of an active vision system). Seven computational requirements were identified: 1) transformation of retinotopic to egocentric mappings, 2) spatial memory for the purposes of medium-term inhibition of return, 3) synchronization of ‘where’ and ‘what’ information from the two visual streams, 4) convergence of top-down and bottom-up information to a centralized point of information processing, 5) a threshold function to elicit saccade action, 6) a function to represent task relevance as a ratio of excitation and inhibition, and 7) derivation of excitation and inhibition values from object-associated feature classes. The model provides further insight into the nature of data representation and transfer between brain regions associated with the vertebrate ‘active’ visual attention system. In particular, the model lends strong support to the functional role of the lateral intraparietal region of the brain as a primary area of information consolidation that directs putative action through the use of a ‘priority map’.

## Introduction

One of the main problems in trying to define the underlying mechanisms of visual attention is that its neurophysiological drive stems from several sources, the weights of which are determined by different contextual paradigms. Visual attention, for example, can be in the context of “searching” or “not searching”, different levels of task-driven information, different levels of task-relevant and object feature complexities or, prior experience or naivety to the visual scene. What is common to all situations, however, is that overt visual attention, through the action of saccade, is essentially a request for further visual information originating from either the same or a different sensory modality system. For example, additional visual data may be required to complete partial peripheral retinotopic information or, to identify objects in relation to associated sound stimuli or, to facilitate appropriate grasping in relation to reach.

One of the main aforementioned context divisions of the operational state of the visual attention system is “searching” or “not searching”. When “not searching”, visual attention is driven predominantly by in-built saliency filters present in the early stages of visual processing within the visual cortex. This “bottom-up” mechanism inherently directs saccades towards objects with certain feature characteristics such as high feature contrast [1] as a result of, for example, either luminance contrast [2], [3], or edge density [4] and temporal features [5]. In the context of “searching”, a further sub-division can be made as to whether this is search within a naive environment (i.e. objects are being viewed for the first time) or the onlooker has experience of the visual scene (i.e. has previously saccaded to objects and features within that scene). Short term visual memory or ‘inhibition of return’ (IOR) is critical within this situation where inhibitory processes mask excitation from previously saccaded to objects [6]. Introducing task and object relevance superimposes another layer of “top-down” bias that can operate at a number of different levels within the system. The first is through a feed-forward pre-attentive priming effect at the stage of early visual processing that biases the strength of specific low resolution peri-foveal visual data [7]. Depending on the complexity of object information required, a request for information is not always generated and the low resolution peri-foveal visual data is sufficient in the context of the task. For example, in a study by Li et al. [8], correct colour and shape of objects within peripheral view could be identified whilst maintaining fixation at the central point. As the complexity of the object feature data increases or rather, the complexity of object feature data required for goal-orientated action increases, the more likely a saccade is required to attain this high resolution, complex feature data. Indeed the complexity of the object feature data can be such that return saccades are required to allow further linking or processing of object features. Here, the system can override IOR mechanisms to facilitate this process [9], [10]. The evidence above strongly demonstrates a high degree of plasticity in the mechanism underlying visual attention that is both cross-modal and adaptable dependent on information held, information required and whether the agent is in a task- directed or task-neutral state. Such a system, therefore, must have the ability to process concurrent and non-concurrent signals from different brain systems that are competing for visual attention and saccade and, thereby provide a neurophysiological forum where both bottom-up and top-down information can be assimilated into a common currency to produce the most appropriate motor response.

Various computational visual attention systems have been constructed in order for robots and other devices to detect and locate regions of interest in their visual world. Such systems often attempt to take account of what is known of the human visual system and employ concepts, such as ‘active vision’, to gain various perceived advantages. However, despite the potential for gaining biological insights from such experiments, the computational requirements for visual attention processing are often not clearly presented from a biological perspective. This was the primary objective of this study, to be attained through two specific phases of investigation: 1) conceptual modeling of a top-down-bottom-up framework through critical analysis of the psychophysical and neurophysiological literature to predict first stage computational requirements, 2) implementation of the model into robotic hardware as a representative of an active vision system and validation through behavioural testing to subsequently identify sec- ond stage computational requirements. Furthermore, critical analysis of the developed model may give the opportunity to derive new hypotheses about the biological system.

For the following discussion, a glossary of terms is presented Table 1.

**Table 1:** Glossary of terms and mathematical variables.

Abbreviation	Explanation
***General***	
FEF	Frontal eye field.
Gaze space	The egocentric space around the whole of the vision system.
ICR	Identified computational requirements.
IOR	Inhibition of return-the inhibition of a saccade to a previously fixated object within a defined time frame.
Linear Ballistic Accumulator	A model to describe the linear accumulation of information to a point of threshold upon which action e.g. saccade is taken. Starting points and rates of accumulation can vary.
LIP	Lateral intraparietal region of the brain predominantly associated with initiation of saccade.
MIP	Medial intraparietal sulcus of the brain predominantly associated with initiation of motor action.
Retinotopic space	The space as currently observed within the camera's visual scene.
what' pathway	Dorsal visual stream that passes through the V1, V2 and V5 layers of the visual arriving at the posterior parietal cortex (and particualrly LIP) and is considered to process and hold spatial information about objects.
where' pathway	Ventral visual strem that passes through V1, V2 and V4 layers of the visual cortex before arriving at the inferior temporal cortex. The ventral visual stream in concerned predominantly with object identification
***Mathematical***	
*E*	Excitatory aspect of task relevance modulation.
*f*	Activation value derived from modulation of the saliency value *s*.
*G_global_*	Set of coordinates associated with egocentric space as a summation of *Glocal* and *G_sm_*.
*G_local_*	Set of coordinates associated with the current retinotopic space.
*G_sm_*	Set of coordinates associated with the spatial memory.
*H*	Inhibitory aspect of task relevance modulation.
*p*	Egocentric co-ordinates derived from retinotopic coordinates modulated by relative and absolute pan and tilt camera positions.
*Q*	The maximum number of attributes that determine the saliency value *s*.
*t*	Time since entry of an activation value f into the gaze space mapping.
*t_*max	The maximum time that a stimulus can be stored in the spatial memory.
*s*	Saliency value derived after visual RGB filters and movement algorithm.
*w*	Bottom-up weightings conferred at the point of initial filtering of visual information.

## Methods

### Stage 1-Model development and first stage computational requirements of the system

The visual cortex operates as a layered (V1–V5) early processing centre of visual information with feedforward and feedback mechanism between layers existing throughout the cortex. Neurons within each layer are tuned specifically to various properties of incoming visual stimuli providing filtering capability for different properties of the incoming visual stimuli for example, contrast ratio, movement, luminance colour, and patterns. The map of the retina appears to be strictly conserved within early visual processing regions (V1–2) and to a lesser degree in subsequent layers (V3–V5) where the visual information becomes more abstracted. Visual information is also anatomically partitioned and functionally dissociated into the dorsal and ventral streams, commonly referred to as the ‘where’/‘action’ and ‘what’/‘perception’ pathways respectively [11], [12].

The dorsal pathway passes through V1, V2 and V5 arriving at the posterior parietal cortex and is considered to process and hold spatial information about objects. The ventral pathway also passes through V1 and V2 but thereafter differs in its route passing through V4 before arriving at the inferior temporal cortex. The ventral visual stream is concerned predominantly with object identification. There has been considerable debate as to why two pathways should have functionally evolved and how, on separating visual data, it can be brought back together to facilitate object-informed motor action. From an evolutionary perspective, it is considered that the required computational transformation differs between the streams. The dorsal stream, in the context of reaching requires precise and egocentric metric transformations in real time whereas the ventral stream is considered to processes object data relative to the scene and in a way that allows comparison with previously stored information about those objects [13]. Although functionally dissociated under the terms ‘action’ and ‘perception’, it is generally considered that both pathways influence motor output, but that the ventral ‘perception’ pathway does so in an indirect manner [14]. It is the dorsal visual stream, however, that appears to predominate during attentional premotor activity and is the considered starting point for both saccade (lateral intraparietal region of intraparietal sulcus [LIP]) and elicited motor action (medial intraparietal sulcus [MIP]) [15].

One of the primary questions that arises from this dichotomy of the visual stream is how the brain manages to maintain a synchronized link between these different forms of visual data? And furthermore, how bottom-up and top-down mechanisms contribute and modulate to this synchronisation process?

**Figure 1:**
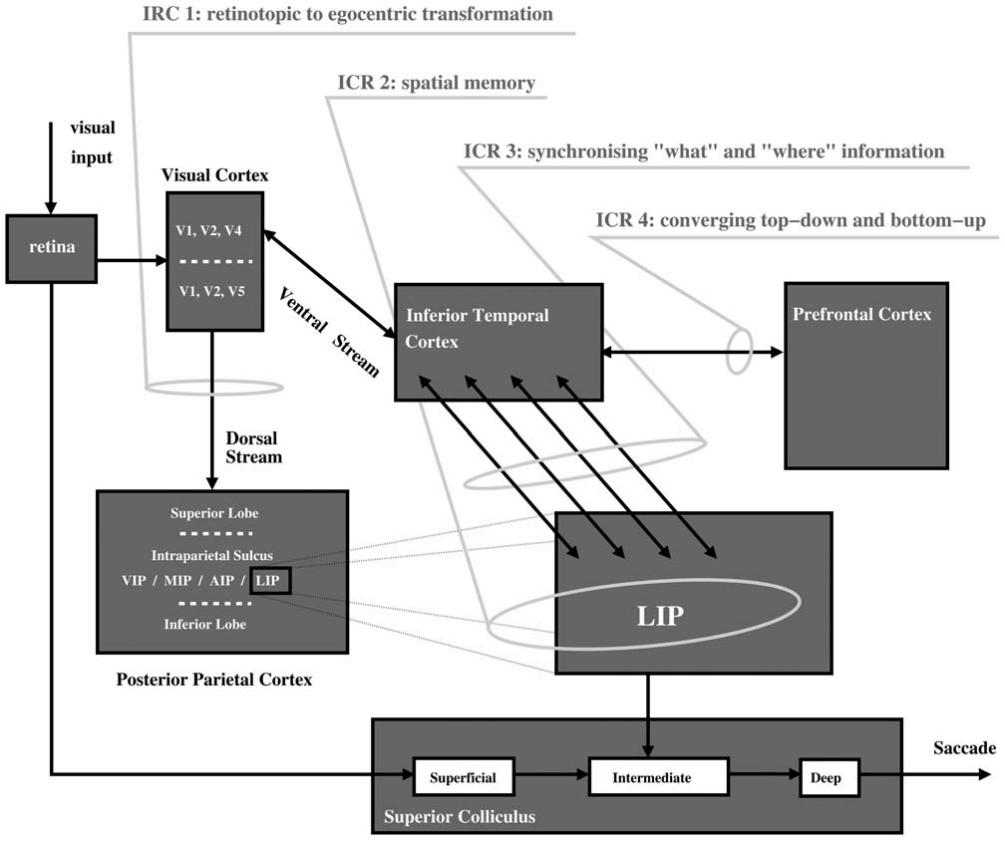
Primary brain regions associated with visual attention with identified first stage computational requirements (ICR) 1–4 (AIP-Anterior Intraparietal region; VIP, Ventral Intra-parietal region; MIP- Medial Intraparietal region; LIP-Lateral Intraparietal region).

In the following, we specify these questions in the form of a biologically-inspired computational model (Figure 1) for overt visual attention integrating bottom-up and top-down mechanisms. This is subsequently used to highlight four primary identified computational requirements (ICR) required to resolve the robotic implementation in a biological plausible way.

#### 1. Egocentric reference frame, ICR 1

The first question that arises in constructing a working architecture for a visual attention system, is the issue of retinotopic versus egocentric mappings. Although described as ‘visual’ attention, vision is not the only driver of saccade with non-vision sensory modalities and internal signals also having this ability. The other critical point here is that saccade is often directed to points outside of the current retinotopic map, thus, there is a requirement to extend this map to incorporate the general egocentric space. This computational requirement is labeled as ICR 1 “retinotopic to egocentric transformation” which in our model occurs along the dorsal pathway (Figure 1). There is strong evidence to suggest that the deep layer of the superior colliculus in the mid-brain, as the near terminal structure in saccade generation, is functional in this respect [16]. Other egocentric maps would, however, be anticipated within higher levels of the brain hierarchy given that a) the conscious perception of space is not limited to a retinotopic framework and b) modulation of putative targets of saccade needs to be carried out within an egocentric framework and it is more probable that this critical function would be carried out within cortical rather than mid-brain structures. Again, the posterior parietal cortex (Figure 1) is a primary candidate brain region in this respect [17].

#### 2. Spatial memory facilitating inhibition of return, ICR 2

Inhibition of return (IOR) refers to the suppression of stimuli (object and events) processing where those stimuli have previously (and recently) been the focus of spatial attention. In this sense, it forms the basis of attentional (and thus visual) bias towards novel objects but more importantly prevents continual fixation on the same highly salient object. It is thus a critical mechanism within any visual attention system. Although the neural mechanism underpinning IOR is not completely understood, it is well established that the dorsal frontoparietal network, including frontal eye field (FEF) and superior parietal cortex are the primary structures mediating its control. These are some of the many modulatory and affecting structures of the deep superior colliculus (optic tectum in non-mammals), the primary motor structure controlling saccade. Although visual information from the retina starts at the superficial superior colliculus, and there are direct connections between the superior and deep layers [18] the former cannot elicit saccade directly [19]. This information has to be subsequently processed by a number of cortical and sub-cortical structures that place it 1) in context of attentional bias within egocentric saliency maps (posterior parietal cortex) [20], 2) the aforementioned IOR [21], 3) overriding voluntary saccades (frontal eye fields) [18] and 4) basal ganglia action selection [22]. Thus, biologically there exists a highly developed, context specific method for facilitating the most appropriate saccade as a form of attention selection. One of the main problems to overcome in constructing an IOR system is the accurate mapping of the retinotopic space to the global egocentric space i.e. foveated objects within a retinotopic map must be logged within a global egocentric map to allow subsequent comparison with peripheral retinotopic information. The lateral intraparietal (LIP) region is the primary candidate brain region for this process, given its aforementioned position in modulating the transfer of visual information from superficial to deep superior colliculus. LIP also appears to be the most pertinent structure in the phenomenon of IOR with strong modulatory connections to the intermediate layer of the superior colliculus [17], [23], [24] and physiological characteristics that strongly correlate with a linear ballistic accumulator model (a linear accumulation to saccade criterion with different rates of accumulation and starting points for each saccade) that is considered to describe the IOR process [25].

#### 3. Synchronisation of “where” and “what” information, ICR 3

Within a situation where an object appearing in the peripheral retina has low intrinsic salience value (luminance, contrast) but is task relevant, a request for saccade will be made by the terminal region of the ventral stream due to partial recognition of the object. The question then is how does initialization of saccade within LIP of the dorsal visual stream, as a result of this information request in the ventral stream, occur? Biological work has yet to elucidate completely how this issue is resolved, however, data does indicate two possible mechanisms. Firstly, connections identified between the two visual pathways [26] may suggest direct binding of the two mapping systems. Alternatively, given the feedforward and recurrent epoch hypothesis of pre-attentive and attentive vision [27], perifoveal object information modulated in the context of task could be passed back to early processing visual centres, to then move forward again along the dorsal stream.

The recent paper by [28], as an extension of earlier work [29], has demonstrated that visual information along the dorsal pathway arrives earlier compared to the ventral equivalent, and thus tends to suggest that the former solution to this binding problem may be the most applicable. However, it may also be that, even through spatial information about an object arrives first, the modulation of this information in the context of low resolution object feature data in relation to task, may still occur through the aforementioned retrograde passage of information.

#### 4. Task modulation of visual attention through a top-down-bottom-up framework, ICR 4

In the context of constructing a top-down-bottom-up framework, several approaches are possible with one of the key questions being, how centralized is the point of information convergence? This question points to our last identified computational requirement, ICR 4. Given the high inter-modulatory nature of the mammalian brain it is unlikely that there is one major epicenter of information processing, however it may be that there are a limited numbers of terminal regions of information processing, centres for final consolidation of information that sit immediately prior to motor output. Several sources of anatomical and neurophysiological data point towards the posterior parietal cortex as being such a centre of convergence and in particular the LIP region.

In addition to what has been discussed about the LIP region in the context of IOR (ICR 2), LIP neurons are also suppressed when in the receptive field of task-irrelevant distractors [30], [31] but activation enhanced when the salience of visual cues is augmented [32], or when the relative subjective ‘desirability’ of the saccade is increased [33]. Moreover LIP neurons experience relative gain when signaling stimuli relevant as compared to irrelevant to the task [34]. Furthermore, LIP neurons are also activated on presentation of task relevant tactile [35] as well as auditory [36] stimuli demonstrating strong cross-modal functionality. Finally, within paradigms considered to demonstrate different weightings of bottom-up versus top-down strategies, LIP has been shown to be activated within both scenarios [7]. Indeed, the term ‘priority map’, as a combination of salience and relevance, has recently emerged to describe the LIP as a meeting point between bottom-up and top-down information potentially within an egocentric reference frame [1] or, as a locus for non-spatial computations affecting the spatial allocation of attention [37]. In conclusion, substantive evidence supports the notion of LIP existing within a centralized position between the dorso-ventral visual stream dichotomy (Figure 1). As such, this may be the centralized region that allows top-down modulation to occur via the ventral visual stream where complex feature data can be detected and biased (IRC 4, Figure 1) before synchronisation of ventral and dorsal information.

#### 5. Summary

In summary, four primary computational requirements appear to be necessary in the first instance to facilitate a basic architecture for visual attention. The following section refines this first-stage model by developing its design for implementation. By making the model concrete in this way we are forced to confront design decisions that will eliminate infeasible or unworkable mechanisms. We describe how these requirements were met in the order of 1) transforming retinotopic to egocentric mappings, 2) spatial memory for the purposes of medium term inhibition of return, 3) synchronizing “where” and “what” information from the two visual streams and 4) converging top-down and bottom-up information to a centralized point of information processing.

### Stage 2- Model implementation

#### 1. Robotic Setup

The vision system consisted of two cameras (Guppy model; Allied Vision Technologies, U.S.A.) mounted in a motorised active vision head (Model TO40 human stereo system; RoboSoft, France). This provides 4 degrees of freedom; pan, tilt and independent camera vergence movement. Only verge and tilt of the one camera (left) were used (2 DOF).

The motors controlling verge and tilt were operated via their absolute target position 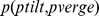 or the change of the current position 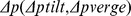, both given in radians (rad). The images generated were RGB at a resolution of 1032×778 pixels. The motors in the Robosoft TO40 pan and tilt system were controlled by an internal controller connected to the main system PC via an ethernet link. The AVT cameras were connected via firewire (IEEE 1394) to the main PC. The software consisted of a linux environment with hardware drivers and bespoke C++ code for the higher level research architecture.

#### 2. Processing RGB data and simulating the dorsal and ventral visual pathways

The image processing of the original RGB camera image data was set up to produce a basic representation of visual input data as seen within vertebrate systems. This was simulated by dividing the original camera RGB data into two data streams; the first fed high resolution image data from a small localised region within the centre of the image (foveal), the second low resolution stream represented visual information outside of this region (peri-foveal). Within this system, peri-foveal information was treated as ‘near-peri-foveal’ or para-foveal in that, as well as being sensitive to object movement, it also contained colour information [8]. The low resolution pixel data were filtered for their content of red (R), green (G) and blue (B) visual information and also for movement 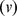
(simple algorithm comparing consecutive image frames) using basic visual software which then produced an intensity value
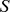
for each of the respective colours. Movement was monitored through a simple algorithm monitoring differences in image frames. This RGB data was then used to generate a final saliency value 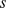 for each pixel as follows:
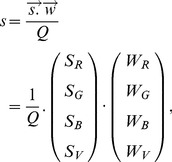
(1)where 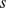 is a real number between 0 and 1, 0.0≤
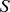
≤ 1.0; 0.0≤
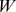
≤ 1.0. 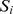 is the filtered colour intensity value and 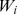 a weighting factor that allows bottom-up bias to be set for individual colours as well as movement. The data was normalised to values between zero and one using the multiplication factor 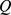 where 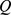 represents the maximum number he scalar product can achieve.

For the high resolution image data (‘what’ pathway), feature filters were applied to extract the exact intensity of each individual colour component and information about shape or texture. This data was summarised as a feature vector, the construction of which will detailed in section 8.

In summary, the original RGB image data was transformed into two data streams: one delivering a low resolution retinotopic map using RGB filtering and the other a high-resolution-based feature vector 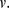 The low resolution retinotopic map represented the dorsal (“where”) stream, while the feature vector derived from the high resolution data at the image centre represented the ventral (“what”) stream.

#### 3. Robotic architecture for visual attention

**Figure 2:**
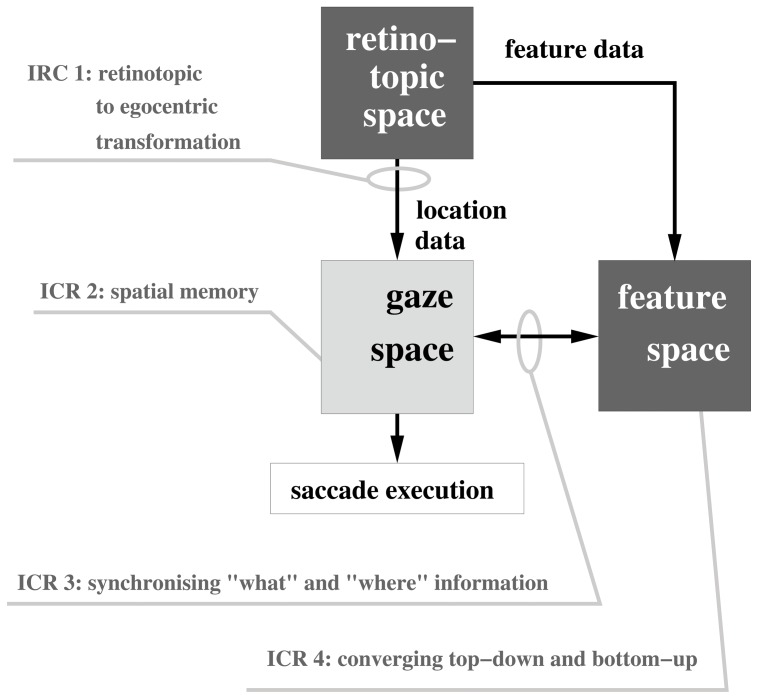
Computational domains of the robotic architecture.

Figure 2 summarizes the data flow between three computational domains of the robotic architecture, alongside the four identified computational requirements, to be discussed in the following sections.

#### 4. Object fixations

In parallel to the biological equivalent, the high resolution feature vector 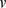 could only be generated once the object was located in the centre of image, mediated via saccadic movements of the active vision system. The method to achieve this has previously been described [38] but in brief, a peripheral stimulus located at a 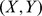
coordinate within the camera's two dimensional visual scene (equivalent to the retinotopic map) was linked, through a previously learned mapping process, to specific relative tilt and verge camera motor movements 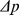 where, these motor movements brought the stimulus to the image centre.

#### 5. Transformation from retinotopic to egocentric coordinates, ICR 1

Central to whole architecture is the transformation of visual information from retinotopic to egocentric co-ordinates. This is critical because it creates a common currency of spatial information to locate objects irrespective of eye, head or arm position. This was carried out by adding the previously described relative verge and tilt motor movements 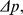 required to saccade to a stimulus at position 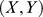 (within the two dimensional visual scene of the camera [retinotopic map]), to the absolute motor positions of the active vision system 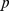 [39]. The stimulus 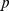
was then stored within the egocentric map (referred to as the gaze space) as the considered representation of the previously described LIP brain structure. The gaze space thus held stimulus 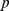
values that were putative targets for saccade.

#### 6. Inhibition of return, ICR 2

Inhibition of return (IOR) was implemented by having stored stimuli in LIP (as a result of saccade) inhibit stimuli of the same corresponding coordinates 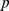
within the retinotopic mapping. The entries into the LIP structure were also set to have a decay value, as a generally accepted characteristic of visual memory [40], and were removed when the decay value reached zero. This allowed the visual system to repeatedly saccade towards a range of salient objects and not get ‘stuck’ on consistently high saliency stimuli.

Up to this stage of the discussion, we have defined the egocentric position of a stimulus 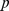 (in terms of relative and absolute verge and tilt motor positions) and allocated to that stimulus a saliency value 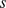 based on a normalised function of its RGB and movement content. The next stage was to categorise saliency values dependent on whether they were located retinotopically or egocentrically. Post-categorisation, these values were referred to as activation values 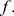 Stimuli that were currently located in the retinotopic map (i.e. present in the current visual field) were stored as activation values 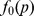 and kept in a set referred to as 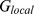 such that
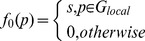
(2)where 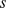 is a real number between 0 and 1, 0.0≤
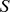
≤1.0.

In other words, the stimulus 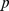
was assigned the activation value 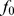 that equaled the saliency value 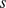 if the 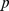 value was part of the 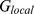 set otherwise the value was held at zero.

**Figure 3:**
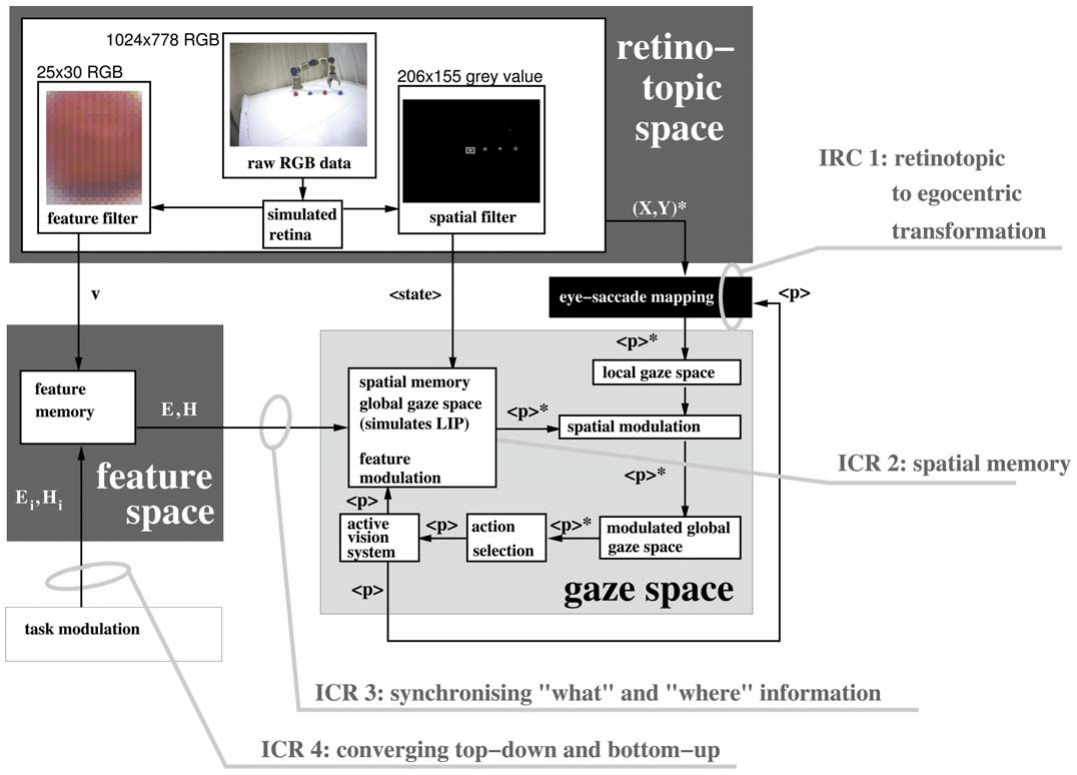
Computational architecture for visual attention integrating bottom-up and top-down modulation.

The spatial memory (Figure 3) also stored 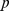
coordinates representing previous saccades. Again these 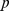
values were kept within a specific set 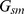 and similarly had activation values 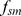. The quality of this value was different from 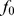 in that it was modulated by a decay function 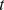 determined by the time passed since 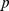 was added to 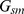:
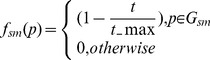
(3)where 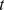
is a natural number between 0 and 1, 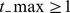, 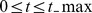, and 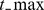
is the maximal time a coordinate is stored in the spatial memory.

In other words, the stimulus 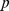 was assigned the activation value 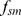 equal to 1 minus the ratio of decay time over the maximal time if the 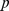 value was part of the 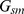 set otherwise, the value was held at zero.

When time 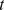 surpassed the maximal time 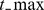, 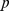 was removed from 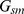. This equated to a standard neurophysiological decay function for stimulus memory [41].

The actual process of IOR was thus achieved through ‘spatial modulation’ of the retinotopic map by the spatial memory (activation values 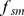 on activation values 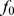). This was done by creating a new set 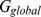 with stimulus 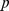 again having a respective activation value 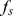, calculated as:
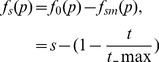
(4)where 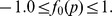


In other words, 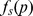, as the subtraction of stored activation values in the spatial memory 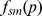 from stimuli currently being observed in the retinotopic map 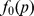, is equivalent to the initial saliency value 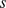 minus the decay function.

**Figure 4:**
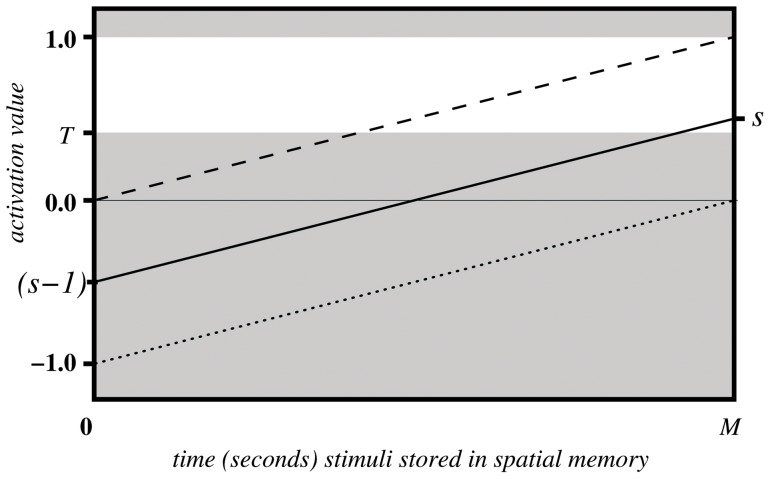
Activation values over time undergoing spatial modulation.

Figure 4 illustrates the linear change of activation values over the time 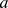 (solid line), if not affected by a re-saccade up to that point. One can see, when the maximal remaining time is reached 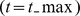 the activation value is back to its original value 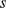. Figure 4 also illustrates the two extreme cases of original saliency value; the dashed line shows the case when the original saliency value is maximal 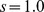, while the dotted line represents minimal saliency values, i.e. 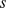 is close to zero.

Having established a mechanism for inhibition of return through the subtraction of one map from another, a criterion for generating a saccade had to be defined. Given the varying and fluctuating nature of the input data, it was decided that a threshold function 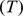 would be the most appropriate and simplest strategy to transform continually modulated input data into motor output and thus action selection. This approach was supported by the biological literature and in particular the IOR data generated from the study by Ludwig et al. [25]. The white region in the Figure 4 indicates the domain of activation values which could trigger a saccade action.

In summary, ICR2 in relation to the biological phenomenon of IOR required 3 layers of map or array with two representing the retinotopic and egocentric mappings and the third being derived from the first two. Within the latter layer, a threshold function was applied for the purposes of saccade and action selection.

#### 7. Synchronization between dorsal and ventral visual stream, IRC 3

The synchronization problem in keeping ‘dorsal’ spatial and ‘ventral’ feature information bound was achieved through a linking function such that each 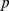 value stored in the spatial memory (as the result of a saccade) pointed to its own feature vector stored in the feature memory (Figure 3). This approach, as previously discussed, represents the direct mapping, biological solution between the two visual streams (Figure 1). The relevance of the object to the task (as determined by its feature vector) altered the activation value of 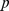 in the global gaze space 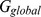 and this constituted the top-down modulation element of the visual attention system. Task relevance was represented by the value 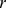 and was constructed through the following formula:
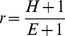
(5)where 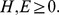



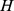
 and 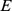 values represented the metric for inhibitory and excitatory modulation respectively based on task relevance. How these values were generated is described in the following section.

Thus, the full modulation of the activation value of 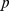 by object features and the decaying IOR function was:
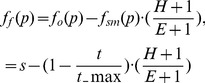
(6)where 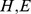 are real numbers and 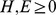.

In other words, the final activation value 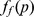 is the original filter-based saliency value 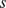 now modulated by the IOR decay function and the task relevance of the stimulus to the task at hand.

**Figure 5:**
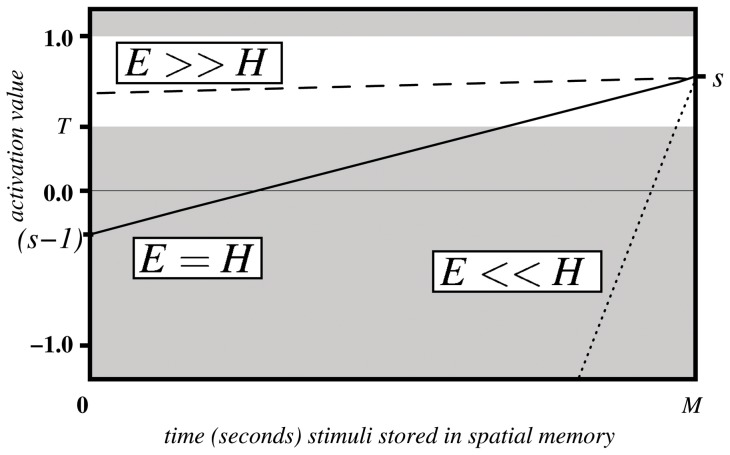
Activation values over time undergoing spatial and feature modulation for different excitation and inhibition levels; 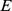 = excitatory modulation, 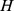 = inhibitory modulation, 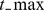 = maximal time a co-ordinate is stored in the spatial memory.

The diagram shown in Figure 5 illustrates the evolution of activation values over time for different parameter settings of 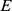 and 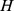
while 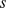 is fixed.

#### 8. Converging bottom-up and top-down information, ICR 4

Once 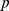 values were assigned to a specific feature vector, given their feature qualities (RGB and movement), this information required processing in the context of task relevance 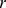 to generate the appropriate modulation factor, as previously described, of:
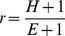
(7)


In this explanation, we assume four disjunct feature classes; red (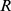), green (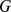), blue (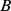) and undefined (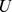). The latter (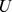) was that which could not be defined by the RGB filter system, for example gray values. Task relevance was defined by the matrix 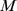 where:
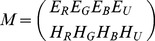
(8)


The classification of the previously described feature vector 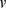 assigned to 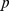 was also expressed in the form of a vector 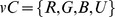. For example, an object classified as red would have the following vector:
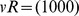



The final E and H value for a particular stimulus 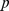 was thus derived by multiplying together the vector with the matrix, such that:
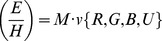
(9)


For example, assuming a setup where there are only red and blue balls present and red balls are task relevant, this would derive an 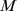 of:
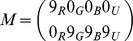



The feature vectors generated as a result of having two balls of different colours would be:
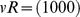
for red objects,



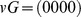
for green objects and



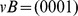
for blue objects.

Applying equation 9, the final excitation and inhibition values for classified elements in the global gaze space are thus:
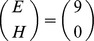
for red objects,



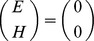
for blue objects and



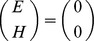
for green objects.

With these parameter values, the points in the spatial memory associated with blue are thus inhibited whilst red stimuli will show higher activation values. Consequently, it is more likely that the system will fixate on red as opposed to blue objects.

It should also be noted at this point that the ratio of 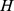 to 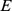 has dramatic effects on the final activation value 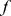. For example, if 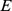 is much greater that 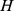, 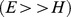 we get high activation values at the beginning and a gradient close to zero, whilst for 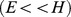, the gradient is large and the initial activation values are below zero (Figure 5). This obviously has a significant effect when activation values reach threshold and thus how the system responds to different objects of differing saliency characteristics.

### Validation

#### 1. Introduction

In this set of experiments we compared fixation patterns towards different coloured objects on a table with different set parameters for bottom-up and top-down modulation. Various weightings 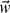 (Eq. 1) of the visual input filters represented different bottom-up input as intrinsic biases towards specific visual properties. For top- down modulation, we fixed the aforementioned weighting 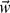 values and tested different excitation 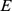 and inhibition 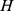 parameters as representations of task relevance.

In all the experiments, the maximum retention time for spatial memory was set at 20 seconds 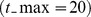, the threshold for triggering eye saccades was fixed at 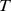 = 0.1 and the recording time was 500 seconds.

The system behaviour was quantified in terms of fixation patterns [42] where the number of saccades and the fixation time (time [sec] between two saccades) were recorded. In addition, for each saccade the 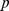 value was also logged along with its corresponding features class. Out of these data were derived the absolute number of saccades, total fixation time and the average fixation time for each object present. Four balls were placed on the table (two red and two blue) and the excitation and inhibition values were pre-defined for each colour class.

As previously described (Eq. 9), the direct feature modulation was as follows:
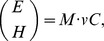


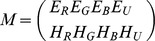
where different excitatory 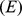 and inhibitory 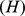 values for red and blue were tested.

**Figure 6:**
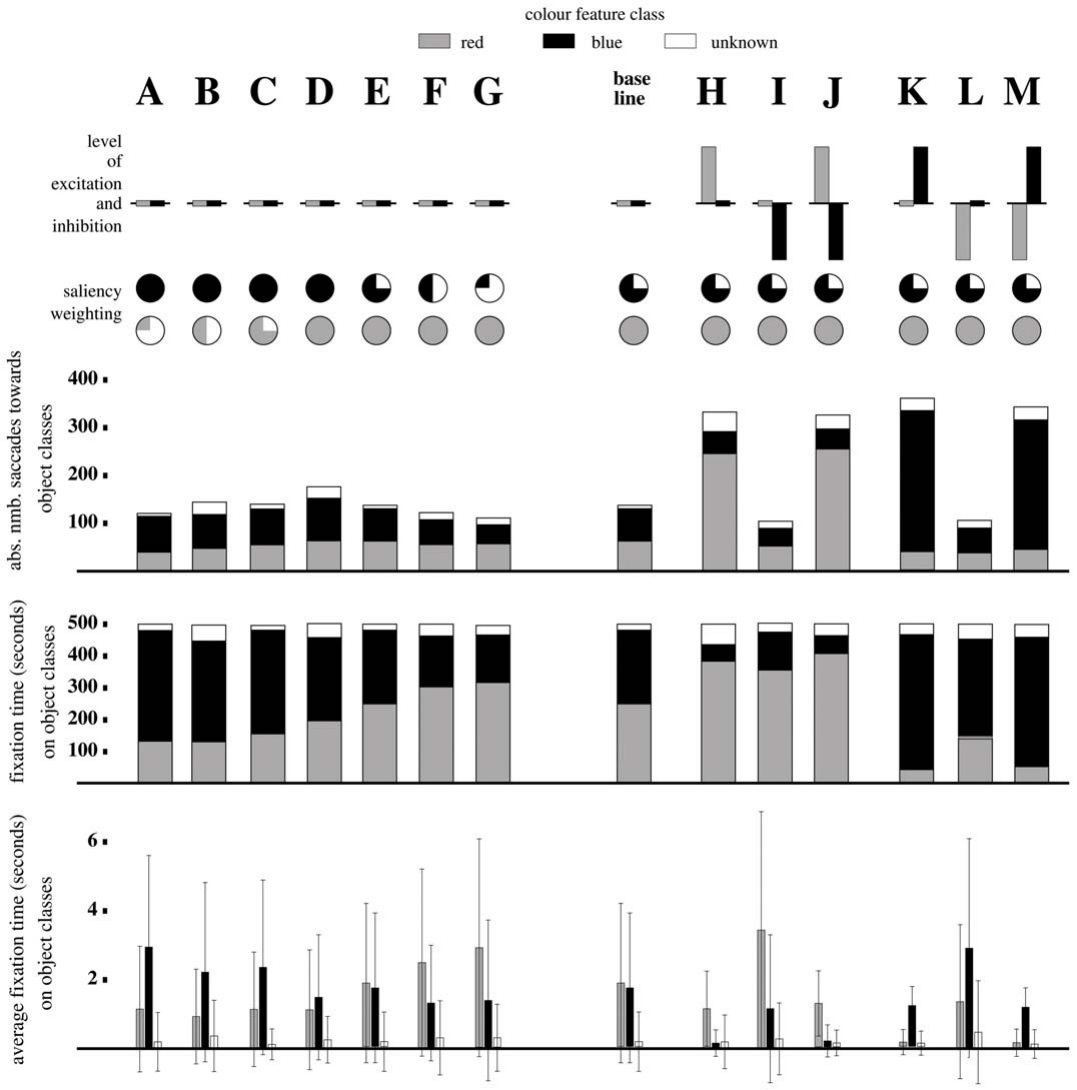
Bottom-up (columns A-G) versus top-down (columns H-M) modulation of visual attention; filled circles refers to bottom-up saliency weightings.

Results are shown in Figure 6 with the data for each parameter setting for 500 seconds summarised in each column.

#### 2. Bottom-up modulation only

Bottom-up modulation only data are presented in columns A to G (Figure 6) with different saliency weightings 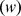 and combination of weightings for colours blue and red for each column. All 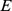 and 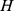 values (top-down modulation) in this instance were set to zero for all colours.

The system performed as was generally expected with increases in weightings 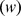 for each colour causing increases in respective total number of saccades, the average fixation time per object and the total fixation time for the colour class. Some exceptions were observed, however, to this general rule that appeared to be explained by a bias to towards the blue colour class. This was most evident when both 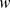 value for blue and red were set to 1 (column D) and all three measures showed a preponderance towards the blue colour class. Similarly for the absolute number of saccades towards red, this only surpassed blue once the latter dropped to a relative weighting of 25%. This effect was difficult to explain and was suspected to be a hardwire bias at the early filtering stage of the system process.

Interestingly on a number of occasions the object did not fall into either colour classification and was recorded as unknown (white labeled regions in Figure 6). This refers to the cases where an object was not completely centered leading to non-RGB colour values and the classification of the feature vector 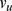 as unknown (colour feature class 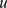).

#### 3. Top-down modulation

Top-down modulation was tested in three different variations that again biased the system towards a particular colour class, either red or blue. As a base line for all the feature modulation experiments, we selected the saliency weighting in column E, 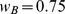 and 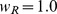. This saliency weighting produced the most balanced response to red and blue objects (number of saccades towards the two colour classes were nearly the same and the average fixation time relatively similar). Implementation of top-down bias was possible in three different ways and each of these were tested for the two different colour classes (red, blue) (Figure 6):

1. excitation only: 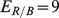 while other 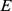 and 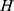 values are zero (columns H and K);
2. inhibition only: 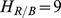 while other 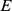 and 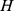 values are zero (columns I and L);
3. excitation and inhibition: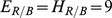 while other 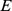 and 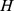 values are zero (columns J and M).

Top-down modulation compared with the base line (bottom-up modulation only) fixation patterns were significantly different (Figure 6). Excitation of a specific feature class (columns H, J, K and M), resulted in a rise in the absolute number of saccades towards objects of that colour class. The total fixation time towards objects associated with the excited colour feature class also increased whilst there was a general trend of decreased mean fixation time for all feature classes observed.

Applying inhibition only (columns I and L) resulted in a decrease in the numbers of saccades towards the inhibited feature class. There was also a reduction in the total fixation time per object class and a lower average fixation time per object, compared to the non-inhibited feature classes. With respect to the baseline, there was no actual change in the total number of saccades, whereas the average fixation time and total fixation time for all colour classes increased. There also appeared to be no noticeable difference in results between implementing excitation only (columns H and K) versus the combined excitation-inhibition strategy (columns J and M).

In comparison to the bottom-up data (changes in 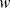), a number of similar data patterns were observed. Top-down modulation via inhibition only (columns I and L) appeared to produce patterns with strong similarities with the two ends of the spectrum of the bottom-up modulation data (columns A and G). In particular, the profile of measures were extremely similar between data sets G and I and A and L where, in the latter, the system is biased towards blue by saliency weighting (A) and via the inhibition of red (L).

To conclude, the system demonstrated several characteristics akin to its biological equivalent. In particular, increasing the saliency of incoming visual stimuli into the system increased the average number of saccades and fixation duration towards the object as has consistently been reported in the biological literature (see [43] for review). Increasing the task relevance of the object had a similar effect [44]. Perhaps more importantly, the system also had the ability to combine both types of bias in additive, inhibitory or competition-based ways that also support the current thinking of how top-down and bottom-up guidance systems of visual attention potentially integrate [45]–[47].

## Discussion

### General comments

Computational models of working brain systems are an extremely important methodological tool in fully understanding the putative functional roles of individual brain regions. Whilst neurophysiological and scanning studies in combination with specific paradigm testing are extremely useful in linking brain regions with certain types of processes, the actual nature of information transfer within these processes is often lacking. Extending the computational model to implementation in hardware has the advantage, as demonstrated within this study, of a) fully validating the system as functional within a bottom-up and top-down framework and b) provoking additional questions about computational requirement associated with embodiment not necessarily considered during the first stage of assessment. Four initial computational requirements were identified during model construction:

1. transforming retinotopic to egocentric mappings (ICR 1);
2. spatial memory for the purposes of medium term inhibition of return (ICR 2);
3. synchronizing ‘where’ and ‘what’ information from the two visual streams (ICR 3);
4. converging top-down and bottom-up information to a centralized point of information processing and (ICR 4).

Three additional computational requirements were identified during the second (implementation) stage of the investigative process:

1. a threshold function, 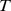;
2. a function representing task relevance as a ratio of excitation and inhibition;
3. deriving 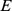 and 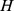 values from object-associated feature classes.

These three additional computational requirements provoke new questions about how the biological system may be working. For example, what is the exact relationship between the threshold function 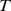 and the underlying action selection process for eye saccades and secondly, is there a linear relationship between task relevance 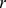 and its modulation of the visual attention system. Also, and in relation to the third additional computational requirement (derivation of the 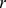 value), the model requires further validation to assess if this framework accommodates additional feature combinations such as tactile feedback during object manipulation. Preliminary studies have generated interesting data [48] but require further analysis and comparison with human data in order to provide scientific insight about multi-modal visual attention mechanisms in biological systems.

### The central role of LIP

The representation of LIP as an egocentric map and a point of information convergence within a bottom-up top-down framework was found to have several computational and implementation advantages. In essence, it allowed objects, held in precise spatial coordinates, to be continually modulated over time by any number of excitatory or inhibitory factors (e.g. IOR, task relevance). This interpretation sits very comfortably with the biological data where LIP activation is gain-modulated dependent on the relevance of incoming visual stimuli to the context of the task [34]. Fundamental to the practical implementation of the system and for the purposes of stimuli eliciting sub- sequent action, was the threshold function 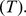 As previously discussed, this function also has strong biological grounding [25] where data generated from IOR paradigms using human subjects closely fitted the threshold-based Linear Ballistic Accumulator model. The point at which motor threshold is reached has often been interpreted as the point at which a “conscious” decision is made to act [49]. This is an attractive interpretation of how LIP may work, as a site of terminal processing and ultimately decision making about motor action. Gottlieb et al., however, have challenged this notion based on their data that have demonstrated LIP activation outside of motor planning or execution [34]. They postulate, in return, that whilst LIP may be convincingly identified as an internal priority map responsible for covert spatial attention, it is not the final stage of eliciting motor action.

### The computational advantage of two visual pathways

The evolutionary and thus functional basis for the bifurcation of visual data into the dorsal and ventral stream has somewhat remained an enigma. However, on implementation of the visual attention system, it became apparent that functionally dividing accurate spatial location data of an object from its task relevance, and to then have the latter modulate the former may be computationally advantageous [13]. In particular, the egocentric reference frame (gaze space), facilitated easy synchronisation of the “what” and “where” information on eye-saccades.

### Top-down modulation

One of the issues that occurred in constructing the visual system within this study, was deciding at what point within the process of visually assessing a naive scene (viewed for the first time) does top-down modulation occur. The review of visual attention systems by Theeuwes [47] suggests that a number of discrete processing stages take place from the start of input of visual information through to the point of saccade. Initially, during the pre-attentive stage, a feed-forward sweep of visual information results in a first stage allocation of attention based solely on the intrinsic saliency characteristics of stimuli within the visual scene. This visual information originates from foveal and non-foveal regions of the retina and thus contains both high and low resolution data. The second stage of processing involves recurrent feedback processing to allow top-down modulation of this incoming visual information. Although this is considered to be the attentive phase of visual attention, it should be noted that this is still modulation of low resolution peripheral retinal data prior to saccade. This system differs from the visual system constructed here in that top-down modulation of incoming stimulus-based salience data can only occur once objects have previously been saccaded to. Within the ‘naive visual scene’ scenario, at the start of image processing within this system, top-down modulation cannot occur because no saccades have yet occurred, compared with task relevance and thus stored in the LIP region. However, once several cycles of image processing and saccades have taken place, then top-down modulation of incoming peripheral visual data is continually occurring. Thus, in the non-naive scenario, the system adheres to the recurrent top-down modulation theory of the incoming visual information. One other difference compared to the biological system can be demonstrated in the work of [47] whereby distractors of high intrinsic salience have the ability to delay reaction times in what is referred to as the additional singleton search task. Within our system, such a test would result in the generation of an overriding saccade away from the correct fixation response as opposed to simply a delay in the correct response. This suggests that within the biological system there is an additional ability of peri-foveal data analysis and subsequent dampening of stimulus if the latter is not relevant to the task. Indeed, recent work by [50] where inversion of a visual image (thus reducing top-down object relevance but maintaining bottom-up object saliency) increased fixation latency, also suggests some analysis of peripheral data in the context of task relevance. Further development of the computational model would need to take this in account.

Lastly, generating the task relevance value for individual objects was achieved through modulation of object feature vectors. In this instance, the model was limited to one object feature (colour) but could have easily been extended. Recent work by [51] proposed a biologically plausible model whereby only task relevant features are extracted (and thus modulated) from the object, reducing the overall computational requirement of the system. This may be a very useful way to extend the current architecture to deal with more complex object tasks.

## Conclusion

The primary objective of this study was to identify the computational pre-requisites of visual attention within an active vision system through model development, implementation and validation within robotic hardware and, in particular, to critically assess how bottom-up and top-down biases could be integrated within one system. The study was successful in this respect, with several computational requirements being identified and with the system behaving and generating fixation data considered reliably representative of the primary characteristics of its biological counterpart. The proposed model therefore provides further insight into the nature of data representation and transfer between brain regions relevant to the vertebrate ‘active’ visual attention system. In particular, the model lends strong support to the functional role of the lateral intraparietal region of the brain as a primary area of information consolidation within egocentric co-ordinates and the idea that it operates within the brain as a priority map in relation to putative action [34].

Furthermore, the model provoked further questions about the functional nature of the biological system, for example, when intrinsic salience of objects are fixed, does task relevance of objects affect attention in a linear fashion? Further psychobiological research has the ability to answer such questions and, through an iterative process of changing the model based on data generated, the opportunity to build a very complete and accurate picture of how integrated bottom-up and top-down modulations may be working within an active visual attention system.
